# A Comparison of Statistical Methods for the Discovery of Genetic Risk Factors Using Longitudinal Family Study Designs

**DOI:** 10.3389/fimmu.2015.00589

**Published:** 2015-11-19

**Authors:** Kelly M. Burkett, Marie-Hélène Roy-Gagnon, Jean-François Lefebvre, Cheng Wang, Bénédicte Fontaine-Bisson, Lise Dubois

**Affiliations:** ^1^Department of Mathematics and Statistics, University of Ottawa, Ottawa, ON, Canada; ^2^School of Epidemiology, Public Health and Preventive Medicine, University of Ottawa, Ottawa, ON, Canada; ^3^Nutrition Sciences Program, University of Ottawa, Ottawa, ON, Canada

**Keywords:** genetic association, family design, longitudinal studies, linear mixed models, generalized estimating equations

## Abstract

The etiology of immune-related diseases or traits is often complex, involving many genetic and environmental factors and their interactions. While methodological approaches focusing on an outcome measured at one time point have succeeded in identifying genetic factors involved in immune-related traits, they fail to capture complex disease mechanisms that fluctuate over time. It is increasingly recognized that longitudinal studies, where an outcome is measured at multiple time points, have great potential to shed light on complex disease mechanisms involving genetic factors. However, longitudinal data require specialized statistical methods, especially in family studies where multiple sources of correlation in the data must be modeled. Using simulated data with known genetic effects, we examined the performance of different analytical methods for investigating associations between genetic factors and longitudinal phenotypes in twin data. The simulations were modeled on data from the Québec Newborn Twin Study, an ongoing population-based longitudinal study of twin births with multiple phenotypes, such as cortisol levels and body mass index, collected multiple times in infancy and early childhood and with sequencing data on immune-related genes and pathways. We compared approaches that we classify as (1) family-based methods applied to summaries of the observations over time, (2) longitudinal-based methods with simplifications of the familial correlation, and (3) Bayesian family-based method with simplifications of the temporal correlation. We found that for estimation of the genetic main and interaction effects, all methods gave estimates close to the true values and had similar power. If heritability estimation is desired, approaches of type (1) also provide heritability estimates close to the true value. Our work shows that the simpler approaches are likely adequate to detect genetic effects; however, interpretation of these effects is more challenging.

## Introduction

It is increasingly recognized that longitudinal studies – where an outcome, or phenotype, is measured on each individual at multiple time points – have great potential to shed light on complex disease mechanisms involving genetic factors. The Quebec Newborn Twin Study (QNTS) is a prospective longitudinal investigation on twin pairs born between April 1, 1995 and December 31, 1998 in the Greater Montreal Area (1). Six hundred and sixty-two [662, 38% monozygotic (MZ)] (67%) of the eligible twin pairs and their families were recruited into the study with 322 pairs undergoing additional laboratory assessments (1–3). To be eligible, the twin pairs had to share the same household environment and be free from major congenital diseases (1). The QNTS collected information on a wide range of biological, cognitive, behavioral, and social factors including anthropometric measures and dietary intake (1). DNA is also available on a subset of the twins and sequencing is in progress. Using results from QNTS, earlier studies have estimated the heritability over time of traits, such as dietary intake, eating behaviors, and body size (2–4).

An important goal of QNTS is to perform longitudinal analysis on associations between genetic factors, such as those from the inflammatory pathway, and developmental phenotypes like obesity. Obesity is characterized by a state of persistent low-grade inflammation (5). Adipose tissue and its infiltrated macrophage and immune cells release proinflammatory mediators, such as tumor necrosis factor-*α* (TNF-*α*), interleukins (IL)-1b and -6, and chemokine ligand 2 (CCL2) (5). Inflammatory cytokines and chemokines activate nuclear transcription factors, such as activator protein-1 (AP-1) and nuclear factor-kB (NF-kB), which in turn induce the expression of proinflammatory genes in a positive feedback manner (5, 6). Caloric and nutrient excess induce hypothalamic inflammatory signals, which, among other effects, induce insulin/leptin resistance (5, 7, 8). Variations in genes related to the leptin signaling pathway have already been implicated in the classical monogenic model of severe obesity (9). In addition, a Swedish cross-sectional study showed that genetic variants of the inflammatory cytokines IL-1*β* and IL-1RN were associated with significant differences in body fat in young men (10). A systematic review performed in 26,944 healthy adults also revealed that haplotypes in the IL-6 gene were associated with increased waist circumference and body mass index (11). Thus, genetic variations influencing the inflammatory response likely affect obesity phenotypes.

Since the QNTS contains longitudinal data on twin pairs, a challenge with the analysis of QNTS data is accounting for the correlation due to both repeated measurements over time and repeated measurements within a family. Many different approaches have been developed to analyze longitudinal data on unrelated samples [reviewed in Ref. (12), for example]. In particular, regression-based methods allow estimation of the effects of covariates of interest while accounting for the correlation of repeated measurements on an individual. In a marginal model approach, the mean of the response is specified with a linear or generalized linear model and the correlation between response values is modeled with a prespecified correlation structure. These models are fit using Generalized Estimating Equations (GEE) (13). In multi-level or hierarchical models, the correlation between the repeated measurements is assumed to be due to unobserved subject-specific regression coefficients that are modeled as random effects in the linear or generalized linear mixed model. These models are fit using maximum likelihood-based approaches (14).

Similarly, many methods exist for the analysis of genetic data collected on families but at a single point of time. Among these methods are those that use random effects to model the correlation among family members based on kinship and include the genotype of interest as a fixed effect in a mixed model (15, 16). Specifically for twin studies, the structural equations classical twin model (17) can also be used to test for genetic association by including the genotype as a covariate in the model.

There has been much interest in evaluating approaches for the analysis of family data measured at multiple time points. For example, multiple Genetic Analysis Workshops have included longitudinal family data for researchers to evaluate and compare performance of different approaches; these contributions are summarized in Gauderman et al. (18), Kerner et al. (19), and Beyene and Hamid (20). However, the evaluated methods have generally involved simplifications to the data structure so that either standard longitudinal methodology or standard family-based methodology can be used.

The analysis strategies developed so far can be broadly classified as (1) two-stage analyses where family-based methods are applied to summaries of the observations over time, (2) longitudinal-based methods that simplify the familial correlation, and (3) family-based methods that simplify the temporal correlation. In the two-stage approaches, repeated time measurements can be collapsed by taking the average over time or the slope, intercept or residuals from regressing each individual phenotype values on time. Methods appropriate to family data are then applied to the collapsed scores [see in Ref. (21, 22), for recent examples]. For methods of type (2), many groups have evaluated either the GEE or GLMM approach to longitudinal analysis when the family structure is simplified. For example, Choi et al. (23), Shi et al. (24), and Sung et al. (25) all include a random family effect in their mixed effect models, implicitly assuming that the correlation between observations from related family members is constant regardless of the relationships. In the GEE approach evaluated by Shi et al. (24) and Sung et al. (25), a compound symmetry correlation structure is assumed for the correlation between family members, which explicitly assumes constant correlation between family members. This assumption is known not to be realistic with, for example, samples of MZ and dizygotic (DZ) twins. MZ twins are more closely related genetically than are DZ twins, and so we would expect that phenotypes measured on MZ twins are more correlated than phenotypes on DZ twins. Finally, the Bayesian approach for variance component estimation with longitudinal family data described by Burton et al. (26) more realistically models the familial correlation between family members due to both shared genes and shared environment. However, although all time points are included in the analysis, the correlation between time points is assumed to be constant. Phenotype values measured at adjacent time points would not be more similar to each other than say the first time point to the last. Again, this is clearly unrealistic. To our knowledge, no approach concurrently models both the correlation due to time and family without simplifying one of these correlation structures in a way known to be unrealistic.

As the three different types of analysis strategies involve three different simplifying assumptions, it is of interest to evaluate the effects of these simplifications. In this paper, we examine the performance of some of the analytical methods described above for investigating associations between genetic factors and longitudinal phenotypes in family data using data simulated based on the QNTS. The family structures, ages, and sex of the subjects are taken from the QNTS. Genotype data were simulated based on these family structures and the BMI phenotype at multiple time points was simulated based on the growth curves of the subjects and under multiple genetic models. As the true models are known, we evaluate which approaches most closely estimate the true parameter values and we estimate the power of each method. As no method proposed to date can fully model the correlational complexity of the longitudinal family design, our work provides important guidance to analysts about when each approximation is expected to perform adequately. Although many different studies compare the performance of some of the methods outlined above [for example, Ref. (23–25)], comparisons are not exhaustive as typically only two methods within each of the three types, for example the marginal to multi-level model, are compared. In addition, we simulated data under multiple different genetic models, and we include the Bayesian approach in our comparison.

## Materials and Methods

We evaluated several different approaches for the analysis of longitudinal family data. In the next section, we describe the methods that we evaluated. We then describe our simulations and our metrics for judging each approach.

### Statistical Approaches

We divide the approaches into three categories (1) those that use a summary statistic of the phenotype over time, (2) those that simplify the correlation due to sampling multiple members of the same family, and (3) methods that simplify the correlation due to repeated measurements over time.

#### Summary Statistic Approach

The simplest form of analysis for a longitudinal outcome measure is to summarize the repeated measures for each individual. The analysis based on the summary measure can then be done using standard methods for family-based designs. We used the following individual summary measures: (1) the average over the time points, (2) the intercept and the slope calculated using a linear regression of BMI on age for each individual, and (3) the area under the curve (AUC) calculated using the trapz R function [pracma package (27)] implementing the trapezoidal rule method. We then analyzed each summary measure using three models. First, we fit a linear mixed model with a random family effect to account for the twin correlation using the lme function [nlme package (28)]. This model simplifies the familial correlation.

Since we are analyzing twin data, we then used the classical twin model (17) as implemented in the twinlm function of the mets package (29), which provides estimates of variance components (additive genetic, shared environmental, and residual) and heritability. The classical twin model is a path model incorporating the different variance components:

$$Y_{ij}=x_{ij}\beta +aA_{ij}+dD_{ij}+cC_{ij}+eE_{ij},$$

where *Y_*ij*_* is the trait value for twin *j* in twin pair *i*, *β* is a vector of fixed effects corresponding to the vector of covariates ***x****_*ij*_* to be included in the model and including an intercept, *a*, *d*, *c*, and *e* are the path coefficients and *A_*ij*_*, *D_*ij*_*, *C_*ij*_*, and *E_*ij*_* are mutually independent with standard normal distributions. The variance components for additive genetic $\left(\sigma_{A}^{2}\right)$, dominance genetic $\left(\sigma_{D}^{2}\right)$, common environmental $\left(\sigma_{C}^{2}\right)$, and residual components $\left(\sigma_{E}^{2}\right)$ are the squares of the path coefficients *a*, *d*, *c*, and *e*, respectively. We fit the “ACE” model, including additive genetic, common environmental and residual variance components (i.e., dropping the dominance term from the model). Heritability can be estimated from this model as the ratio $\sigma_{A}^{2}/(\sigma_{A}^{2}+\sigma_{C}^{2}+\sigma_{E}^{2})$.

For all methods, covariates in the linear model included sex and genotype.

#### Simplifying Familial Correlation

We then considered methods that simplify the familial correlation. In the case of twin data, this means that the correlation structure corresponding to twin zygosity (MZ and DZ) is not correctly modeled. With the simplified correlation structure, conventional statistical methods for longitudinal data can be used. We evaluated two models described in Choi et al. (23). The first is a marginal GEE model for which we used three correlation structures: exchangeable, autoregressive, and unstructured. The second model is a three-level, hierarchical (subject-specific) model. These models were fit with the R gee (30) and nlme packages, respectively. For both the GEE and hierarchical approaches, covariates in the linear model included sex, genotype, age, and genotype-by-age, depending on the underlying genetic model.

#### Simplifying Correlation Over Time

A third strategy involves modeling the correlation of phenotype that is due to kinship, as in the approaches of type (1). However, rather than use a summary of all time measurements, each time point is included in the analysis and the correlation between time points is assumed to be due to kinship. A Bayesian-based approach to estimate the variance components that could handle arbitrary family structure and continuous and binary traits was proposed by Burton et al. (26) and implemented using WinBUGS (31). This approach was not presented as a means of detecting association with genetic factors; however, association can be assessed by including genotypes at measured SNPs in the linear model.

We now describe our Bayesian model, which includes both genotype and genotype-by-age effects and assumes the twin study design. We assume *n* twin pairs are measured at six time points. Let the trait value for individual *j* of twin pair *i* at age *k* be denoted by *Y_*ijk*_* for i=1,…,n; j=1,2; k=1,…,6. Letting *G_*ij*_* and *S_*ij*_* represent the genotype (coded assuming an additive allelic effect) and sex of the individual *j* in twin pair *i*, and *T_*ijk*_* represent the age at the *k*th time point, the full model describing the association between trait and covariates is

$$\mu_{ijk}=(\beta_{0}+\beta_{0ij})+\beta_{S}S_{ij}+\beta_{G}G_{ij}+(\beta_{T}+\beta_{Tij})\times T_{ijk}+\beta_{GT}G_{ij}\times T_{ijk}$$

$$Y_{ijk}\sim N\left(\mu_{ijk},\sigma_{E}^{2}\right),$$

where the regression parameters *β*_0_, *β_*S*_*, *β_*G*_*, *β_*T*_*, and *β_*GT*_* are all considered fixed effects, *β*_0_*_*ij*_* is the random intercept, and *β_*Tij*_* is the random time effect. $\sigma_{E}^{2}$ is the residual variance.

The random effects are further decomposed into a random effect due to kinship and a random effect due to common environment. Letting MZ and DZ represent MZ and DZ twins, respectively, the model for the random intercepts for the *i*th twin pair is

$$\beta_{0i}\sim N\left(0,V_{0}\right),V_{0}=\left[\begin{array}{cc} \sigma_{A}^{2}+\sigma_{C}^{2} & \{\begin{array}{c} \sigma_{A}^{2}+\sigma_{C}^{2}\text{ if MZ}\\ \frac{1}{2}\sigma_{A}^{2}+\sigma_{C}^{2}\text{ if DZ} \end{array}\\ \{\begin{array}{c} \sigma_{A}^{2}+\sigma_{C}^{2}\text{ if MZ}\\ \frac{1}{2}\sigma_{A}^{2}+\sigma_{C}^{2}\text{ if DZ} \end{array} & \sigma_{A}^{2}+\sigma_{C}^{2} \end{array}\right]$$

and the model for the random slopes for the *i*th twin pair is

$$\beta_{\text{Ti}}\sim N\left(0,V_{T}\right),V_{T}=\left[\begin{array}{cc} \tau_{A}^{2}+\tau_{C}^{2} & \{\begin{array}{c} \tau_{A}^{2}+\tau_{C}^{2}\text{ if MZ}\\ \frac{1}{2}\tau_{A}^{2}+\tau_{C}^{2}\text{ if DZ} \end{array}\\ \{\begin{array}{c} \tau_{A}^{2}+\tau_{C}^{2}\text{ if MZ}\\ \frac{1}{2}\tau_{A}^{2}+\tau_{C}^{2}\text{ if DZ} \end{array} & \tau_{A}^{2}+\tau_{C}^{2} \end{array}\right]$$

where ***V*_0_** is the covariance matrix for the random intercept effects and ***V_T_*** is the covariance matrix for the random slope effect. The variance components accounting for correlation due to kinship, the additive polygenic variances are $\sigma_{A}^{2}$ and $\tau_{A}^{2}$ for the intercepts and slopes, respectively. Similarly, the variance components due to common environment are $\sigma_{c}^{2}$ and $\tau_{c}^{2}$ for the intercepts and slopes. The variance components for the intercepts are assumed to be unrelated to those of the slopes. As in the classical twin model, heritability can be estimated as the ratio $\sigma_{A}^{2}/(\sigma_{A}^{2}+\sigma_{C}^{2}+\sigma_{E}^{2})$.

To implement this model, we used the twin model parameterization described in Visscher et al. (32), which is equivalent to the model given above. This parameterization is a twin model version of the parameterization used by Burton et al. (26) and Burton et al. (33). Briefly, this parameterization gives $\beta_{oi}$ and $\beta_{Ti}$ as a sum of random effects corresponding to the twin pair and to the individual. For MZ twins, the individual effect (*Ind*) is shared between the two twins and can be combined with the pair effect (*Pair*), which leads to the following model

$$\beta_{oij}=\;\{\begin{array}{c} {\text{Pair}}_{0i}^{M}\text{ if MZ}\\ {\text{Pair}}_{0i}^{D}+{\text{Ind}}_{0ij}\text{ if DZ} \end{array}$$

$$\beta_{Tij}=\;\{\begin{array}{c} {\text{Pair}}_{Ti}^{M}\text{ if MZ}\\ {\text{Pair}}_{Ti}^{D}+{\text{Ind}}_{Tij}\text{ if DZ} \end{array}$$

where ${\text{Pair}}_{0i}^{M}\sim N\left(0,\sigma_{A}^{2}+\sigma_{C}^{2}\right),{\text{Pair}}_{0i}^{D}\sim N\left(0,\frac{1}{2}\sigma_{A}^{2}+\sigma_{C}^{2}\right)\;$, and Ind_*0ij*_ $∼N\left(0,\frac{1}{2}\sigma_{A}^{2}\right)$. The random time effects are defined similarly, with variance components $\tau_{A}^{2}$ and $\tau_{C}^{2}$ in place of $\sigma_{A}^{2}$ and $\sigma_{C}^{2}$, respectively. Burton et al. (33) mention that this parameterization would be expected to assist convergence.

As this is a Bayesian approach, prior distributions are required for all model parameters. Following Burton et al. (26), flat priors are used for all parameters. The prior distributions for the fixed effect slope parameters are assumed to be $N(0,10^{6})$ and the priors for the variance components are all assumed to be Uniform (0,100). During testing, three chains of 10,000 iterations and 1000 burn-in were run and results were compared between chains. Final results for a single dataset were based on one chain of 20,000 iterations, which took ~5 min to run.

### Simulations

**Figure 1:**
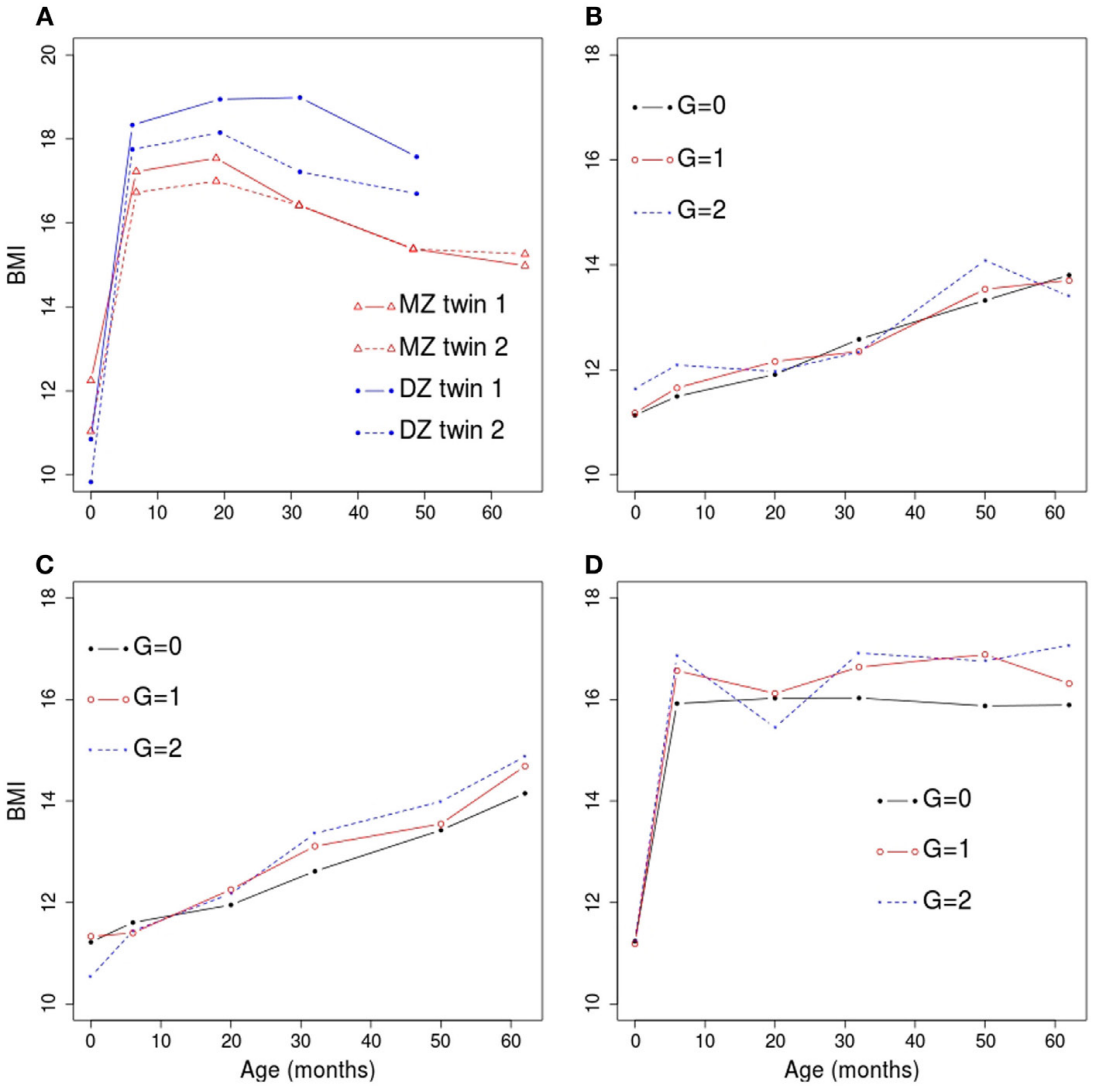
**Example trajectories of observed and simulated BMI versus age in months**. **(A)** Trajectories observed for two twin pairs in the QNTS. The monozygotic (MZ) twin pair is shown in red and the dizygotic (DZ) twin pair is shown in blue. **(B)** BMI trajectories in a simulated dataset under genetic model 2. The sample average at each age within each genotype category (G; coded as 0, 1, or 2 for the number of minor allele) is shown. **(C)** BMI trajectories in a simulated dataset under genetic model 3. **(D)** BMI trajectories in a simulated dataset under genetic model 4.

Our simulations were performed using the model described in Section “Simplifying Correlation Over Time” above, to which we added a fixed effect $\beta_{T^{\prime }}$ to allow modeling of a segmented effect of time with a knot at 6 months of age, so that:

$$\begin{array}{c} \mu_{ijk}=(\beta_{0}+\beta_{0ij})+\beta_{S}S_{ij}+\beta_{G}G_{ij}+(\beta_{T}+\beta_{Tij})\times T_{ijk}+\beta_{T'}\times {T^{\prime }}_{ijk}+\beta_{GT}G_{ij}\times T_{ijk} \end{array}$$

where ${T^{\prime }}_{ijk}=0$ for *k* ≤ 2 and ${T^{\prime }}_{ijk}=T_{ijk}-6$ for *k* > 2. We considered several values of the simulation parameters, as described in Table 1. These values were chosen in order to reflect the observed QNTS data. For example, the segmented effect of time was based on catch-up in growth observed after birth and the magnitude of the age, sex, and genotype effects were based on BMI trajectories observed in QNTS (example trajectories are shown in Figure 1A). We used correlation patterns in the QNTS DZ and MZ twins to define the covariance parameters. Figures 1B–D show examples of simulated average BMI trajectories for selected models.

**Table 1:** **Simulation models**.

Parameter	Genetic effect modeled								
	(1) None	(2) Effect on average				(3) Effect on change (linear)		(4) Effect on change (segmented)	
		(a)	(b)	(c)	(d)	(a)	(b)	(a)	(b)
$\beta_{0}$	11	11	11	11	11	11	11	11	11
$\beta_{S}$	0.5	0.5	0.5	0.5	0.5	0.5	0.5	0.5	0.5
$\beta_{G}$	0	0.1	0.15	0.2	0.3	0	0	0	0
$\beta_{T}$	0.04	0.04	0.04	0.04	0.04	0.04	0.04	0.8	0.8
$\beta_{T^{\prime }}$	0	0	0	0	0	0	0	−0.8	−0.8
$\beta_{GT}$	0	0	0	0	0	0.005	0.01	0.005	0.01
$\sigma_{A}^{2}$	4.5	4.3	4.3	4.3	4.3	3	3	3	3
$\sigma_{C}^{2}$	2.25	2.25	2.25	2.25	2.25	1.5	1.5	1.5	1.5
$\tau_{A}^{2}$	0	0	0	0	0	0.001	0.001	0.001	0.001
$\tau_{C}^{2}$	0	0	0	0	0	0.001	0.001	0.001	0.001
$\sigma_{E}^{2}$	2.25	2.25	2.25	2.25	2.25	1.5	1.5	1.5	1.5

We simulated 788 children (226 DZ pairs and 168 MZ pairs) based on the number of QNTS twins with at least three time points available. Sex and age values were defined as observed in the QNTS data. In order to get a complete dataset with six time points per individual, we imputed each missing visit (age) value by randomly sampling an existing age value at that visit. We chose to perform simulations on a complete dataset to facilitate the comparison of methods (see Discussion). Genotype data were simulated by assigning genotypes to parents for a single nucleotide polymorphism (SNP) with minor allele frequency of 0.3 and using Mendelian transmission to obtain the genotypes of the DZ twins and one MZ twin per family. The second MZ twin was obtained by duplicating the first twin’s genotype. Genotypes were coded as 0, 1, or 2 for the number of minor alleles. We considered four different scenarios for the genetic effect (see Table 1): (1) no genetic effect (used to estimate the type I error of the genetic association test), (2) effect on average BMI values, (3) effect on BMI change with linear effect of time, and (4) effect on BMI change with segmented effect of time. Values of the phenotype were simulated according to each simulation model using R and the rmvnorm function of the mvtnorm package. For all models the heritability resulting from the specified simulation parameters is ~0.5. We based the choice of all simulation parameters on the observed QNTS data. We performed 2000 replicates of each simulation model.

All analyses were performed in R (34) except where otherwise noted. Simulation model 4 was analyzed as simulation model 3, thus ignoring the segmented effect of time. We calculated the mean and SD of the parameter estimates over the 2000 replicates. For the methods of type (1) and (2), which are all frequentist approaches, we estimated power or type I error as the percentage of replicates for which the *p*-value of the genotype effect, or the genotype–time interaction effect when included, was smaller than 0.05. For the Bayesian approach, our estimate of power is the percentage of replicates where the 95% credible interval excluded 0.

## Results

**Figure 2:**
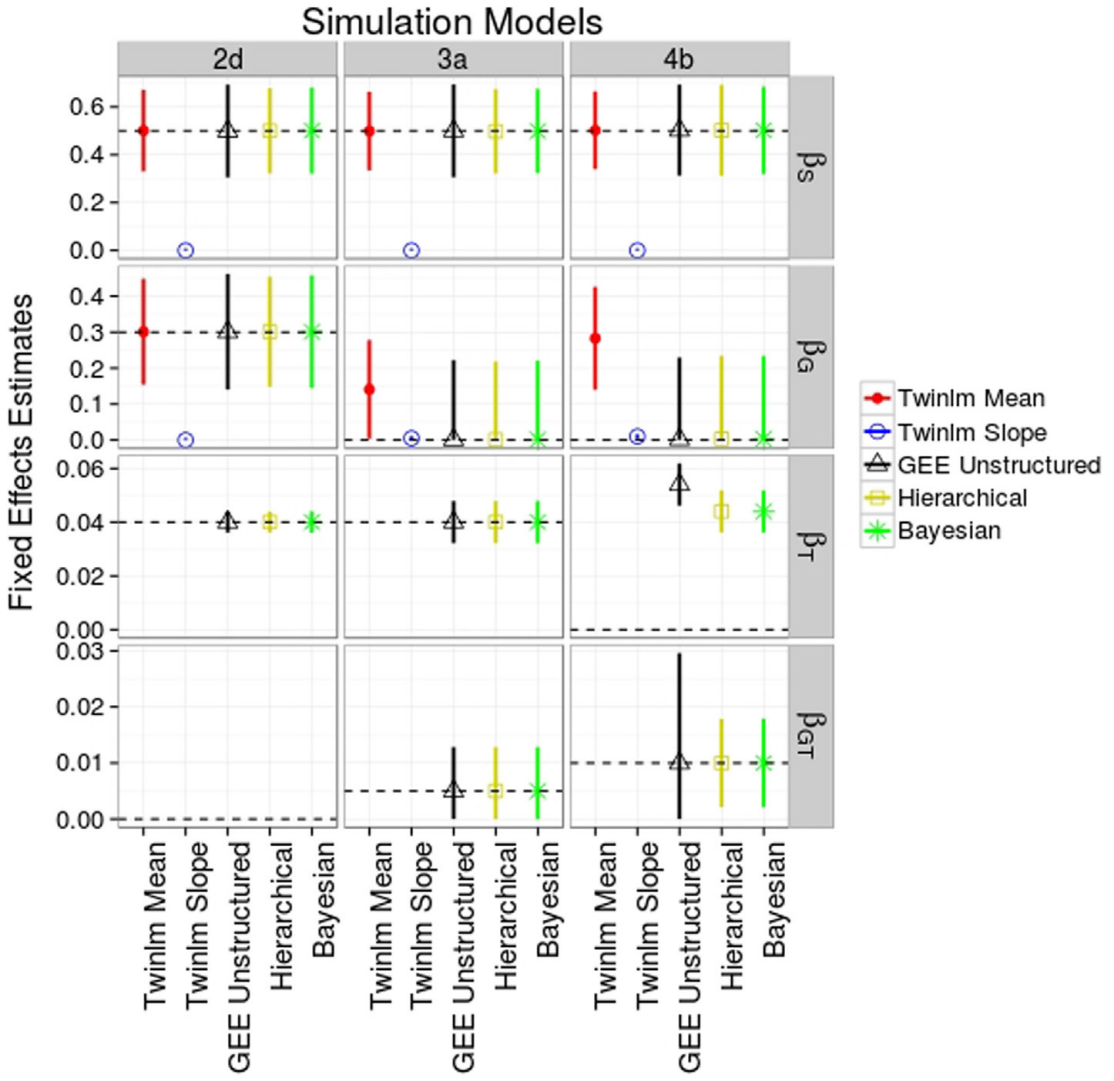
**Mean estimated values (SD) for fixed effects from selected simulation and analysis models**. Analysis models shown are the classical twin analysis of the mean (twinlm mean in red) and slope (twinlm slope in blue), the marginal GEE model with unstructured working correlation matrix (GEE unstructured in black), the three-level hierarchical model (Hierarchical in yellow), and the Bayesian approach (Bayesian in green). The effect of genotype on rate of change (genotype–time interaction) was not modeled when not simulated.

Figure 2 shows the average across the 2000 simulations of the parameter estimates for the fixed effects for selected genetic models and analysis methods; Table S1 in Supplementary Material shows the averages for all genetic models. Among the summary statistics, the AUC is harder to interpret in terms of actual differences in BMI. Unlike the mean and slope summary statistics, the change in the AUC associated with the genetic effect (as estimated by the regression coefficient) cannot easily be interpreted in terms of BMI values. Moreover, the AUC analysis did not provide more power to detect genetic effects compared to other approaches (data not shown); we have, therefore, omitted the results of the AUC phenotype analyses. Similarly, the analysis using the intercept as phenotype did not provide more information about the genetic effect and had lower power than the mean (data not shown), and we, therefore, also do not discuss this analysis further.

In general, all methods of analysis yielded accurate estimates for most of the fixed effects of main interest (the main and interaction genetic effects; see Figure 2; Table S1 in Supplementary Material). For genetic model 2d, all analysis methods except the classical twin model with the slope phenotype gave point estimates of the genetic effect very close to the true value of 0.3. However, since the genetic effect was simulated to be on the mean of the phenotype we would not expect the analysis on the slope phenotype to produce accurate estimates. Similar results were seen for models 2a–c (Table S1 in Supplementary Material).

When the genotype affected the rate of change (models 3a and 4b shown in Figure 2), this effect was picked up by the main genetic effect in the classical twin analysis of the mean phenotype since the model cannot include a genotype-by-age term; therefore, the point estimate for the main genetic effect would not be expected to be close to the true value. In the classical twin analysis of the slope phenotype, the genotype–age interaction is captured by the main effect due to SNP and the parameter estimates are very close to the parameter value for the genotype–age interaction terms. Results were similar for models 3b and 4a (Table S1 in Supplementary Material).

In terms of the point estimates of the fixed effects, there was little difference between the marginal and hierarchical models (Figure 2; Table S1 in Supplementary Material) for all genetic models. We ran the marginal model analysis with three different working correlation matrices (exchangeable, autoregressive, and unstructured); since results were similar between these analyses (data not shown) we only present the analysis using the unstructured correlation matrix. The point estimates for the Bayesian approach are also close to the true values (Figure 2; Table S1 in Supplementary Material).

**Figure 3:**
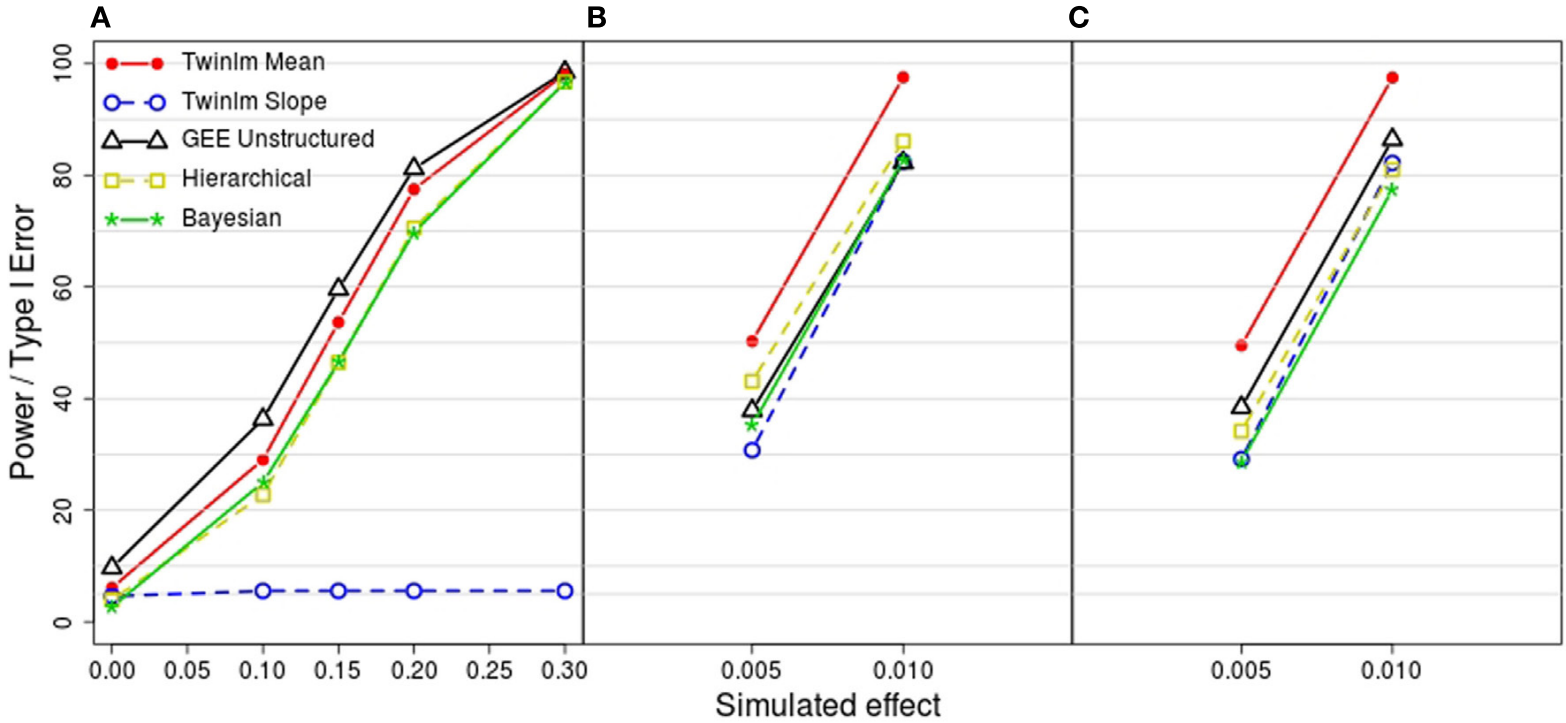
**Estimated power or type I error from the 2000 simulated datasets for each model**. **(A)** Estimated type I error from model 1 (simulated genetic effect at 0) and estimated power to detect a simulated genetic effect on the mean phenotype of 0.1, 0.15, 0.2, and 0.3 from model 2. **(B)** Estimated power to detect a simulated linear genetic effect on the rate of change of 0.005 and 0.01 from model 3. **(C)** Estimated power to detect a simulated segmented genetic effect on the rate of change of 0.005 and 0.01 from model 4. Methods shown are the classical twin analysis of the mean (twinlm mean in red) and slope (twinlm slope in blue), the marginal GEE model with unstructured working correlation matrix (GEE unstructured in black), the three-level hierarchical model (Hierarchical in yellow) and the Bayesian approach (Bayesian in green).

Figure 3 shows the estimated power and type I error rates for the methods shown in Figure 2. Type I error (shown in Figure 3A when the simulated effect is 0 in simulation model 1) was well controlled except when the familial correlation was simplified (analysis of summary measures with a single random family effect and GEE models), where the type I error rates ranged from 7.5 to 10% (results not shown except for GEE with unstructured correlation in Figure 3). Power was similar across methods. For genetic model 2, there was very low power to detect the genetic effect with the slope phenotype; this reflects the fact that these models were simulated so that the genetic effect was on the mean. Notably, the classical twin model with the mean phenotype had power close to maximal for genetic model 2 and the highest power for genetic models 3 and 4, even though these models were simulated so that the genetic effect was on the rate of change and not on the mean.

Variance components partitioning genetic, environmental and residual variance are estimated by two of the tested methods: the classical twin analysis and the Bayesian model. The classical twin analysis of the mean summary statistic provided accurate estimates of variance components and heritability of the mean (over time) phenotype (for model 1: simulated *h*^2^ = 0.5, estimated *h*^2^ = 0.50 ± SD = 0.103; results were similar for the other models and for the slope phenotype, Table S2 in Supplementary Material, and also for different heritability values, Table S3 in Supplementary Material). Estimates of variance components by the Bayesian model were very poorly estimated (Table S4 in Supplementary Material); we discuss this further below.

## Discussion

In this work, we have described the performance of multiple approaches to analyse longitudinally collected family data in estimating the effect of a common genetic variant. Although methods have been developed to handle the correlation due to repeated measurements over time of longitudinal designs and other methods have been developed that model the correlation due to kinship in family designs, the methods proposed for studies that involve both types of correlation have required a simplification of either the time or the family structure that *a priori* is known to not be realistic. Nevertheless, if interest is focused on the estimation of the main or interaction fixed effects and the power to detect a genetic effect, we found that all methods performed quite well even with an incorrectly modeled correlation structure. Interestingly, power remained high for all approaches even when the linear model used in the analysis was different from the true underlying model (Model 4, segmented time effect). As it is challenging to implement methods with more complicated correlation structures, such as the approach of Burton et al. (26), our work shows that the estimation methods offered by standard software like R or SAS are likely to be adequate for most models of association between genetic factor and phenotype.

The most straightforward approach for the analysis of longitudinal family data is to use a summary statistic for each individual as the phenotype in a family-based analysis. We evaluated four summary statistics – the mean, the intercept and slope of a regression of each individual phenotype on age, and the AUC – and we highlighted results for the mean and the slope. We found that even under the model where the genetic effect was on the slope of the phenotype over time (gene-by-age interaction), the power to detect the genetic effect stayed high with the mean phenotype analysis. The main limitation in using summary measures is that it is difficult to clearly distinguish between a genetic effect on the mean phenotype or on the rate of change in the phenotype since the mean also captures the effect on change.

Since we were analyzing twin data, we evaluated the performance of summary measures mainly with the classical twin analysis as implemented in twinlm. A benefit to twinlm is the ability to estimate the variance components; a disadvantage is that this software is only applicable to twin family structures. Therefore, with other family structures a general pedigree approach, such as that implemented in ASSOC of SAGE (16) would be required. A related approach to summary measures is the two-stage approach of Hossain and Beyene (35), which tests for a genetic effect on the residuals of a mixed model accounting for familial correlation. This approach would be preferred if the speed of computation, such as with GWAS data, is a concern.

Although generally power was similar and the genetic fixed effects were well estimated by all methods, not all methods provide estimates of the additive genetic variance. This estimate is important if heritability estimation is one of the goals of the analysis. Estimation of variance components parameters is done using twinlm and the Bayesian approach (discussed below). For the standard longitudinal approaches, the GEE approach treats the variance as a nuisance parameter and is not recommended if the variance components are of direct interest. The multi-level modeling does provide variance estimates but the variance that is estimated is not the additive genetic variance because of the simplification of the family structure. It may be possible to extend to longitudinal data existing mixed model parametrization of the biometrical twin models [e.g., in Ref. (36)], although fitting these models may be difficult.

For our simulated data, we assumed no missing data. Specifically, all individuals in the dataset were observed at exactly six time points. We assumed an equal number of observations per individual to simplify the scripting of the Bayesian approach described in Burton et al. (26); however, this assumption is not realistic when analyzing real data and ideally methods should be able to handle missing time points. For the summary statistic approaches, if values are missing at some time points, one could use either imputation or compute the summaries with the missing values omitted. The latter could lead to higher variability of the collapsed values if the summaries are based on very different numbers of observations for the different individuals. The longitudinal regression-based methods can be run with unequal numbers of observations per individual and the Bayesian approach could also be scripted to handle unequal numbers.

Because we were interested in comparing the Burton method to the other methods, we chose to implement the approach with the same non-informative prior distributions. Burton et al. (26) justify their selection by noting that with the large sample sizes used (50 families of size 5) there is likely little sensitivity to the choice of the prior distribution. Our simulated datasets of 394 twins are also of large sample size. We evaluated sensitivity to the choice of prior distribution by running Models 1–4 with different prior distributions on five randomly sampled datasets. For the fixed effects, we altered the variance parameter of the Normal prior from flat (10^6^) to more informative (100 and 500). For the variance components, we modified the uniform range to include negative values and also to be less wide [*U*(−10,10) and (−10,50)]. The results are essentially unchanged (results not shown).

The Burton approach seemed promising as it was able to handle the correlation due to family structure, variance components are estimated, it is flexible enough to allow more complicated models and family structures, and all time points were simultaneously analyzed. However, there are a number of potential issues with using this approach. First, though all time points are included in the analysis, the correlation between time points is assumed to be due to the family structure. This implies that phenotype values measured at adjacent time points are no more correlated than those measured at distant time points, an assumption that is clearly not realistic. Burton et al. (26) do address this shortcoming but highlight that to model the additional correlation over time would make the approach susceptible to poor MCMC mixing and convergence. Convergence issues like this are a common and important criticism of MCMC approaches. Second, although WinBUGS or OpenBUGS (37) provide powerful tools for Bayesian analysis, they are not necessarily user-friendly. Implementation of the hierarchical Bayesian models described here is not straightforward for the beginner or novice WinBUGS user. To overcome these scripting challenges, we assumed equal number of observations per individual and no missing data; however, in practice, the script should be flexible enough to handle both which would increase the complexity of the scripting. Finally, the variance components were not well estimated by this approach, unlike in Burton et al. (26). This could be due to the fact that our model was based on different family structures (twins only) or that there may have been convergence issues associated with our model parameterization. Indeed, traceplots of multiple runs on a single dataset did show that the genetic and environmental variance parameters appeared to mix poorly, which in practice makes results from this model questionable even though we observed that the fixed effects were well estimated. Modifying the prior distribution did not improve estimation (results not shown). A multivariate normal parameterization of the random effects could be evaluated as a means to improve results; however, *a priori* it might be expected to perform poorly (33). Finally, it is possible that the convergence issue is related to the Gibbs sampling approach. With Gibbs sampling, each parameter is updated individually, conditional on the other parameters. A disadvantage to this type of sampling is that the current values of the variance components could greatly constrain the proposal distribution for the variance component being updated, especially since the structure of the hierarchical model is such that the total variation of the data is a sum of each of the variance components. Thus, an updating mechanism that jointly proposed new values for all variance components may perform better. Implementing this approach could require programming a different version of this sampler outside of WinBUGS or OpenBUGS, which again greatly limits the use of this approach and requires a data analyst comfortable with this type of specialized programming.

We have presented a review and evaluation of different approaches to the analysis of longitudinal family data. The complexity with analyzing these data is that there are two sources of correlation between the observed values: the correlation due to repeated observations within the same family and the correlation due to repeated observations from an individual over time. Other sources of correlation are common in health data. For example, rather than having observations clustered within families, observations can be clustered within hospitals. The equal correlation within group assumption of the GEE and multi-level modeling approaches described would probably be a reasonable assumption, and certainly a more realistic one than making this assumption on family members. Studies also commonly collect multiple phenotype or response variables, such as systolic and diastolic blood pressure, on each subject. Often these are analyzed separately, but we would expect results to be correlated since the phenotypes themselves are correlated. Therefore, a combined analysis might be preferable, particularly if a covariate is thought to be associated with multiple phenotypes (if the covariate of interest is genetic, this is called a pleiotropic effect). Suo et al. (38) provide a recent review and evaluation of different analysis approaches to detect pleiotropic effects, which include MANOVA and data reduction using principal component analysis, for the case of unrelated samples and observations collected at a single time point. It would be interesting to evaluate analysis strategies for multiple phenotypes collected in a longitudinal family study and whether standard approaches with simplified correlation structures work well, as we observed here, or whether more complicated methodology, such as the recently proposed Bayesian latent variable method for longitudinal and family correlation (39), is required.

## Author Contributions

KB and MHRG designed the study, performed part of the programming and data analysis, supervised the simulations and analyses, and wrote the manuscript. JFL ran the simulations and also performed part of the programming and data analysis. LD, BFB, and CW provided content knowledge and wrote part of the manuscript. All authors reviewed and approved the manuscript.

## Conflict of Interest Statement

The authors declare that the research was conducted in the absence of any commercial or financial relationships that could be construed as a potential conflict of interest.

## References

[1] Boivin M Brendgen M Dionne G Dubois L Pérusse D Robaey P The Quebec Newborn Twin Study into adolescence: 15 years later. Twin Res Hum Genet (2013) 16:64–9. 10.1017/thg.2012.129 23200437

[2] Dubois L Diasparra M Bédard B Kaprio J Fontaine-Bisson B Pérusse D Gene-environment contributions to energy and macronutrient intakes in 9-year-old children: results from the Quebec Newborn Twin Study. Physiol Behav (2013) 119:30–7. 10.1016/j.physbeh.2013.05.039 23748099

[3] Dubois L Diasparra M Bédard B Kaprio J Fontaine-Bisson B Tremblay R Genetic and environmental influences on eating behaviors in 2.5- and 9-year-old children: a longitudinal twin study. Int J Behav Nutr Phys Act (2013) 10:134. 10.1186/1479-5868-10-134 24313977 PMC4029536

[4] Dubois L Girard M Girard A Tremblay R Boivin M Pérusse D. Genetic and environmental influences on body size in early childhood: a twin birth-cohort study. Twin Res Hum Genet (2007) 10:479–85. 10.1375/twin.10.3.479 17564506

[5] Gregor MF Hotamisligil GS Inflammatory mechanisms in obesity. Annu Rev Immunol (2011) 29:415–45. 10.1146/annurev-immunol-031210-101322 21219177

[6] Johnson AR Milner JJ Makowski L. The inflammation highway: metabolism accelerates inflammatory traffic in obesity. Immunol Rev (2012) 249:218–38. 10.1111/j.1600-065X.2012.01151.x 22889225 PMC3422768

[7] Jin C Flavell RA. Innate sensors of pathogen and stress: linking inflammation to obesity. J Allergy Clin Immunol (2013) 132:287–94. 10.1016/j.jaci.2013.06.022 23905917

[8] Kälin S Heppner FL Bechmann I Prinz M Tschöp MH Yi CX. Hypothalamic innate immune reaction in obesity. Nat Rev Endocrinol (2015) 11:339–51. 10.1038/nrendo.2015.48 25824676

[9] Waalen J. The genetics of human obesity. Transl Res (2014) 164:293–301. 10.1016/j.trsl.2014.05.010 24929207

[10] Strandberg L Lorentzon M Hellqvist A Nilsson S Wallenius V Ohlsson C Interleukin-1 system gene polymorphisms are associated with fat mass in young men. J Clin Endocrinol Metab (2006) 91:2749–54. 10.1210/jc.2005-2786 16636119

[11] Qi L Zhang C van Dam RM Hu FB. Interleukin-6 genetic variability and adiposity: associations in two prospective cohorts and systematic review in 26,944 individuals. J Clin Endocrinol Metab (2007) 92:3618–25. 10.1210/jc.2007-0877 17623760

[12] Burton P Gurrin L Sly P. Extending the simple linear regression model to account for correlated responses: an introduction to generalized estimating equations and multi-level mixed modelling. Stat Med (1998) 17:1261–91. 10.1002/(SICI)1097-0258(19980615)17:11<1261::AID-SIM846>3.0.CO;2-Z 9670414

[13] Liang K-Y Zeger SL Longitudinal data analysis using generalized linear models. Biometrika (1986) 73:13–22. 10.1093/biomet/73.1.13

[14] Laird NM Ware JH. Random-effects models for longitudinal data. Biometrics (1982) 38:963–74. 10.2307/2529876 7168798

[15] Boerwinkle E Chakraborty R Sing CF. The use of measured genotype information in the analysis of quantitative phenotypes in man. I. Models and analytical methods. Ann Hum Genet (1986) 50:181–94. 10.1111/j.1469-1809.1986.tb01037.x 3435047

[16] George VT Elston RC. Testing the association between polymorphic markers and quantitative traits in pedigrees. Genet Epidemiol (1987) 4:193–201. 10.1002/gepi.1370040304 3609719

[17] Neale M Maes H Methodology for Genetic Studies of Twins and Families. B.V. Dordrecht: Kluwer Academic Publishers (2004).

[18] Gauderman WJ Macgregor S Briollais L Scurrah K Tobin M Park T Longitudinal data analysis in pedigree studies. Genet Epidemiol (2003) 25(S1):S18–28. 10.1002/gepi.10280 14635165

[19] Kerner B North KE Fallin MD. Use of longitudinal data in genetic studies in the genome-wide association studies era: summary of group 14. Genet Epidemiol (2009) 33:S93–8. 10.1002/gepi.20479 19924713 PMC2913696

[20] Beyene J Hamid JS. Longitudinal data analysis in genome-wide association studies. Genet Epidemiol (2014) 38:S68–73. 10.1002/gepi.21828 25112192

[21] Eu-ahsunthornwattana J Howey RA Cordell HJ. Accounting for relatedness in family-based association studies: application to genetic analysis workshop 18 data. BMC Proc (2014) 8(Suppl 1):S79. 10.1186/1753-6561-8-S1-S79 25519407 PMC4143672

[22] Tan Q Hjelmborg JVB Thomassen M Jensen AK Christiansen L Christensen K Hierarchical linear modeling of longitudinal pedigree data for genetic association analysis. BMC Proc (2014) 8(Suppl 1):S82. 10.1186/1753-6561-8-S1-S82 25519411 PMC4144324

[23] Choi Y-H Chowdhury R Swaminathan B. Prediction of hypertension based on the genetic analysis of longitudinal phenotypes: a comparison of different modeling approaches for the binary trait of hypertension. BMC Proc (2014) 8(Suppl 1):S78. 10.1186/1753-6561-8-S1-S78 25519406 PMC4143688

[24] Shi G Rice TK Gu CC Rao DC. Application of three-level linear mixed-effects model incorporating gene-age interactions for association analysis of longitudinal family data. BMC Proc (2009) 3(Suppl 7):S89. 10.1186/1753-6561-3-s7-s89 20018085 PMC2795992

[25] Sung YJ Simino J Kume R Basson J Schwander K Rao DC. Comparison of two methods for analysis of gene-environment interactions in longitudinal family data: the Framingham heart study. Front Genet (2014) 5:9. 10.3389/fgene.2014.00009 24523728 PMC3906599

[26] Burton PR Scurrah KJ Tobin MD Palmer LJ. Covariance components models for longitudinal family data. Int J Epidemiol (2005) 34:1063–77. 10.1093/ije/dyi069 15831561

[27] Borchers HW pracma: Practical Numerical Math Functions. R Package Version 1.8.3 (2015). Available from: http://CRAN.R-project.org/package=pracma

[28] Pinheiro J Bates D DebRoy S Sarkar D The R Core Team nlme: Linear and Nonlinear Mixed Effects Models. R Package Version 3.1-121 (2015). Available from: http://CRAN.R-project.org/package=nlme

[29] Holst KK Scheike T mets: Analysis of Multivariate Event Times. R Package Version 1.1.1 (2015). Available from: http://CRAN.R-project.org/package=mets

[30] Carey VJ Lumley T Ripley B Gee: Generalized Estimation Equation Solver. R Package Version 4.13-19 (2015). Available from: http://CRAN.R-project.org/package=gee

[31] Lunn DJ Thomas A Best N Spiegelhalter D WinBUGS – a Bayesian modelling framework: concepts, structure, and extensibility. Stat Comput (2000) 10:325–37. 10.1023/A:1008929526011

[32] Visscher PM Benyamin B White I. The use of linear mixed models to estimate variance components from data on twin pairs by maximum likelihood. Twin Res (2004) 7:670–4. 10.1375/1369052042663742 15607018

[33] Burton PR Tiller KJ Gurrin LC Cookson WO Musk AW Palmer LJ. Genetic variance components analysis for binary phenotypes using generalized linear mixed models (GLMMs) and Gibbs sampling. Genet Epidemiol (1999) 17(2):118–40. 10.1002/(SICI)1098-2272(1999)17:2<118::AID-GEPI3>3.3.CO;2-M 10414556

[34] R Core Team. R: A Language and Environment for Statistical Computing. Vienna: R Foundation for Statistical Computing (2014).

[35] Hossain A Beyene J. Analysis of baseline, average, and longitudinally measured blood pressure data using linear mixed models. BMC Proc (2014) 8(Suppl 1):S80. 10.1186/1753-6561-8-S1-S80 25519409 PMC4143715

[36] Rabe-Hesketh S Skrondal A Gjessing HK. Biometrical modeling of twin and family data using standard mixed model software. Biometrics (2008) 64:280–8. 10.1111/j.1541-0420.2007.00803.x 17484777

[37] Thomas A O’Hara B Ligges U Sturtz S Making BUGS open. R News (2006) 6:12–7.

[38] Suo C Toulopoulou T Bramon E Walshe M Picchioni M Murray R Analysis of multiple phenotypes in genome-wide genetic mapping studies. BMC Bioinformatics (2013) 14:151. 10.1186/1471-2105-14-151 23639181 PMC3655878

[39] Xu L Craiu RV Derkach A Paterson AD Sun L. Using a Bayesian latent variable approach to detect pleiotropy in the genetic analysis workshop 18 data. BMC Proc (2014) 8(Suppl 1):S77. 10.1186/1753-6561-8-S1-S77 25519405 PMC4143687

