# Supplementary material for: A Comparison of Statistical Methods for the Discovery of Genetic Risk Factors Using Longitudinal Family Study Designs
Source: Front Immunol. 2015 Nov 19;6:589. doi: 10.3389/fimmu.2015.00589 (PMC4652172; doi:10.3389/fimmu.2015.00589)
Supplement: Supplementary file 1 [file supplementary_tables.docx]

***Supplementary Material***

**A comparison of statistical methods for the discovery of genetic risk factors using longitudinal family study designs**

**Kelly M. Burkett^1,*,†^, Marie-Hélène Roy-Gagnon^2,*,†^, Jean-François Lefebvre^2^, Cheng Wang^2^, Bénédicte Fontaine-Bisson^3^, Lise Dubois^2^**

1. Department of Mathematics and Statistics, University of Ottawa, Ottawa, Ontario, Canada

2. School of Epidemiology, Public Health and Preventive Medicine, University of Ottawa, Ottawa, Ontario, Canada

3. Nutrition Sciences Program, University of Ottawa, Ottawa, Ontario, Canada

† These authors contributed equally to this work.

*** Correspondence:**

Kelly Burkett, Department of Mathematics and Statistics, University of Ottawa, 585 King Edward Street, Room 205D, Ottawa, Ontario, K1N 6N5, Canada. [kburkett@uottawa.ca](mailto:kburkett@uottawa.ca)

Marie-Hélène Roy-Gagnon, School of Epidemiology, Public Health and Preventive Medicine, University of Ottawa, 600 Peter Morand Cres, Room 101E, Ottawa, Ontario, K1G 5Z3, Canada. [marie.roy-gagnon@uottawa.ca](mailto:marie.roy-gagnon@uottawa.ca)

1. **Supplementary Figures and Tables**

## Supplementary Tables

**Supplementary Table 1.** Mean estimated values (standard deviation) for fixed effects from selected simulation and analysis models

|  | Model parameters | | | |
| --- | --- | --- | --- | --- |
| Model 2a | $\beta_{S}$ = 0.5 | $\beta_{G}$ = 0.1 | $\beta_{T}$ = 0.04 | $\beta_{GT}$ = 0 |
| Classical twin analysis of mean | 0.501 (0.087) | 0.102 (0.075) | NA | NA |
| Classical twin analysis of slope | 0 (0.004) | 0 (0.003) | NA | NA |
| Marginal model with GEE^a^ | 0.499 (0.099) | 0.101 (0.082) | 0.040 (0.002) | NA^b^ |
| Hierarchical (3-level) model | 0.500 (0.091) | 0.101 (0.078) | 0.040 (0.002) | NA^b^ |
| Bayesian approach | 0.501 (0.092) | 0.102 (0.080) | 0.040 (0.002) | NA^b^ |
|  |  |  |  |  |
| Model 2b | $\beta_{S}$ = 0.5 | $\beta_{G}$ = 0.15 | $\beta_{T}$ = 0.04 | $\beta_{GT}$ = 0 |
| Classical twin analysis of mean | 0.501 (0.087) | 0.152 (0.075) | NA | NA |
| Classical twin analysis of slope | 0 (0.004) | 0 (0.003) | NA | NA |
| Marginal model with GEE^a^ | 0.499 (0.099) | 0.151 (0.082) | 0.040 (0.002) | NA^b^ |
| Hierarchical (3-level) model | 0.500 (0.091) | 0.151 (0.078) | 0.040 (0.002) | NA^b^ |
| Bayesian approach | 0.501 (0.092) | 0.152 (0.080) | 0.040 (0.002) | NA^b^ |
|  |  |  |  |  |
| Model 2c | $\beta_{S}$ = 0.5 | $\beta_{G}$ = 0.2 | $\beta_{T}$ = 0.04 | $\beta_{GT}$ = 0 |
| Classical twin analysis of mean | 0.501 (0.087) | 0.202 (0.075) | NA | NA |
| Classical twin analysis of slope | 0 (0.004) | 0 (0.003) | NA | NA |
| Marginal model with GEE^a^ | 0.499 (0.099) | 0.201 (0.082) | 0.040 (0.002) | NA^b^ |
| Hierarchical (3-level) model | 0.500 (0.091) | 0.201 (0.078) | 0.040 (0.002) | NA^b^ |
| Bayesian approach | 0.499 (0.090) | 0.201 (0.079) | 0.040 (0.003) | NA^b^ |
|  |  |  |  |  |
| Model 2d | $\beta_{S}$ = 0.5 | $\beta_{G}$ = 0.3 | $\beta_{T}$ = 0.04 | $\beta_{GT}$ = 0 |
| Classical twin analysis of mean | 0.501 (0.087) | 0.301 (0.075) | NA | NA |
| Classical twin analysis of slope | 0 (0.004) | 0 (0.003) | NA | NA |
| Marginal model with GEE^a^ | 0.499 (0.099) | 0.301 (0.082) | 0.040 (0.002) | NA^b^ |
| Hierarchical (3-level) model | 0.500 (0.091) | 0.301 (0.078) | 0.040 (0.002) | NA^b^ |
| Bayesian approach | 0.501 (0.092) | 0.301 (0.080) | 0.040 (0.002) | NA^b^ |
|  |  |  |  |  |
| Model 3a | $\beta_{S}$ = 0.5 | $\beta_{G}$ = 0 | $\beta_{T}$ = 0.04 | $\beta_{GT}$ = 0.005 |
| Classical twin analysis of mean | 0.499 (0.084) | 0.141 (0.070) | NA | NA |
| Classical twin analysis of slope | 0 (0.004) | 0.005 (0.003) | NA | NA |
| Marginal model with GEE^a^ | 0.500 (0.099) | 0.001 (0.113) | 0.040 (0.004) | 0.005 (0.004) |
| Hierarchical (3-level) model | 0.498 (0.090) | 0.001 (0.111) | 0.040 (0.004) | 0.005 (0.004) |
| Bayesian approach | 0.499 (0.090) | 0.001 (0.112) | 0.040 (0.004) | 0.005 (0.004) |

^a^ Using an unstructured working correlation matrix. ^b^ Effect of genotype on rate of change (genotype-time interaction) was not modeled when not simulated.

**Supplementary Table 1 (continued).** Mean estimated values (standard deviation) for fixed effects from selected simulation and analysis models

| Model 3b | $\beta_{S}$ = 0.5 | $\beta_{G}$ = 0 | $\beta_{T}$ = 0.04 | $\beta_{GT}$ = 0.01 |
| --- | --- | --- | --- | --- |
| Classical twin analysis of mean | 0.500 (0.083) | 0.283 (0.073) | NA | NA |
| Classical twin analysis of slope | 0 (0.004) | 0.010 (0.003) | NA | NA |
| Marginal model with GEE^a^ | 0.499 (0.100) | 0.003 (0.117) | 0.040 (0.004) | 0.010 (0.004) |
| Hierarchical (3-level) model | 0.500 (0.090) | 0.003 (0.116) | 0.040 (0.004) | 0.010 (0.004) |
| Bayesian approach | 0.501 (0.088) | 0.003 (0.117) | 0.040 (0.004) | 0.010 (0.004) |
|  |  |  |  |  |
| Model 4a | $\beta_{S}$ = 0.5 | $\beta_{G}$ = 0 | $\beta_{T}$ = 0.8 | $\beta_{GT}$ = 0.005 |
| Classical twin analysis of mean | 0.502 (0.083) | 0.142 (0.073) | NA | NA |
| Classical twin analysis of slope | 0 (0.004) | 0.005 (0.003) | NA | NA |
| Marginal model with GEE^a^ | 0.503 (0.097) | 0.002 (0.116) | 0.054 (0.004) | 0.005 (0.005) |
| Hierarchical (3-level) model | 0.502 (0.097) | 0.003 (0.118) | 0.044 (0.004) | 0.005 (0.004) |
| Bayesian approach | 0.502 (0.090) | 0.003 (0.118) | 0.044 (0.004) | 0.005 (0.004) |
|  |  |  |  |  |
| Model 4b | $\beta_{S}$ = 0.5 | $\beta_{G}$ = 0 | $\beta_{T}$ = 0.8 | $\beta_{GT}$ = 0.01 |
| Classical twin analysis of mean | 0.502 (0.083) | 0.283 (0.073) | NA | NA |
| Classical twin analysis of slope | 0 (0.004) | 0.010 (0.003) | NA | NA |
| Marginal model with GEE^a^ | 0.503 (0.097) | 0.002 (0.116) | 0.054 (0.004) | 0.010 (0.010) |
| Hierarchical (3-level) model | 0.502 (0.097) | 0.003 (0.118) | 0.044 (0.004) | 0.010 (0.004) |
| Bayesian approach | 0.502 (0.093) | 0.003 (0.118) | 0.044 (0.004) | 0.010 (0.004) |

^a^ Using an unstructured working correlation matrix. ^b^ Effect of genotype on rate of change (genotype-time interaction) was not modeled when not simulated.

**Supplementary Table 2.** Mean values (standard deviation) for the variance components and heritability estimates from the classical twin model implemented in the twinlm R function

|  | Variance components^*^ | | | Heritability^**^ |
| --- | --- | --- | --- | --- |
| Model 1 | $\sigma_{A}^{2}$ = 4.5 | $\sigma_{C}^{2}$ = 2.25 | $\sigma_{E}^{2}$ = 2.25 |  |
| Mean | 0.7458 (0.1569) | 0.3712 (0.1472) | 0.3744 (0.0401) | 0.501 (0.103) |
| Slope | 0.0015 (3e-04) | 7e-04 (3e-04) | 7e-04 (1e-04) | 0.507 (0.105) |
|  |  |  |  |  |
|  | $\sigma_{A}^{2}$ = 4.3 | $\sigma_{C}^{2}$ = 2.25 | $\sigma_{E}^{2}$ = 2.25 |  |
| Model 2a |  |  |  |  |
| Mean | 0.7098 (0.1593) | 0.3754 (0.1487) | 0.3755 (0.0406) | 0.486 (0.108) |
| Slope | 0.0014 (3e-04) | 8e-04 (3e-04) | 8e-04 (1e-04) | 0.489 (0.108) |
| Model 2b |  |  |  |  |
| Mean | 0.7099 (0.1594) | 0.3753 (0.1487) | 0.3755 (0.0406) | 0.487 (0.108) |
| Slope | 0.0014 (3e-04) | 8e-04 (3e-04) | 8e-04 (1e-04) | 0.489 (0.108) |
| Model 2c |  |  |  |  |
| Mean | 0.7099 (0.1593) | 0.3752 (0.1489) | 0.3758 (0.0422) | 0.486 (0.108) |
| Slope | 0.0014 (3e-04) | 8e-04 (3e-04) | 8e-04 (1e-04) | 0.488 (0.108) |
| Model 2d |  |  |  |  |
| Mean | 0.7097 (0.1594) | 0.3753 (0.1488) | 0.3757 (0.0415) | 0.486 (0.108) |
| Slope | 0.0014 (3e-04) | 8e-04 (3e-04) | 8e-04 (1e-04) | 0.488 (0.108) |
|  |  |  |  |  |
|  | $\sigma_{A}^{2}$ = 3, $\tau_{A}^{2}$ = 0.001 | $\sigma_{C}^{2}$ = 1.5, $\tau_{C}^{2}$ = 0.001 | $\sigma_{E}^{2}$ = 1.5 |  |
| Model 3a |  |  |  |  |
| Mean | 0.7116 (0.1263) | 0.4626 (0.134) | 0.251 (0.0273) | 0.500 (0.089) |
| Slope | 0.0016 (3e-04) | 0.0011 (3e-04) | 5e-04 (1e-04) | 0.503 (0.084) |
| Model 3b |  |  |  |  |
| Mean | 0.7089 (0.1265) | 0.4685 (0.131) | 0.2504 (0.0272) | 0.497 (0.089) |
| Slope | 0.0016 (3e-04) | 0.0011 (3e-04) | 5e-04 (1e-04) | 0.501 (0.084) |
| Model 4a |  |  |  |  |
| Mean | 0.7088 (0.1265) | 0.4767 (0.1316) | 0.2504 (0.0272) | 0.495 (0.088) |
| Slope | 0.0016 (3e-04) | 0.0012 (3e-04) | 5e-04 (1e-04) | 0.498 (0.083) |
| Model 4b |  |  |  |  |
| Mean | 0.7088 (0.1265) | 0.4769 (0.1316) | 0.2504 (0.0272) | 0.494 (0.088) |
| Slope | 0.0016 (3e-04) | 0.0012 (3e-04) | 5e-04 (1e-04) | 0.498 (0.083) |

^*^ Note that the total variances of the mean and slope phenotypes are, respectively, $[{n_{t}{(\sigma}_{A}^{2}+\sigma_{C}^{2}+\sigma_{E}^{2})+(\tau_{A}^{2}+\tau_{C}^{2})\sum_{i=1}^{n_{t}} {{age}_{i}}^{2}]}/{n_{t}^{2}}$ and ${[{(\sigma}_{A}^{2}+\sigma_{C}^{2}+\sigma_{E}^{2})\sum_{i=1}^{n_{t}} \left( {{age}_{i}-\bar{age})}^{2}+(\tau_{A}^{2}+\tau_{C}^{2})\sum_{i=1}^{n_{t}} ({{age}_{i}-\bar{age})}^{2}{age}_{i}^{2} \right]}/{{[\sum_{i=1}^{n_{t}} \left( {{age}_{i}-\bar{age})}^{2} \right]}^{2}},$where $n_{t}=6$ and $\bar{age}$ is the individual average of the age values at the 6 time points. The expected variance, and variance components, of the mean and slope phenotypes can be estimated using the vector of average age values at the 6 time points: (0, 6.3, 19.5, 31.7, 49.7, 62.2). Note that the additive genetic and common environmental variance components include both *σ*’s and *τ*’s since the mean and slope are individual summary statistics over time. Estimates of the variance components and heritability are close to the expected values.

^**^ Heritability = ${\sigma_{A}^{2}}/{{(\sigma}_{A}^{2}+\sigma_{C}^{2}+\sigma_{E}^{2})}$. “True” heritability resulting from simulation parameters is approximately 0.5 for all models.

**Supplementary Table 3.** Mean values (standard deviation) for the variance components and heritability estimates from the classical twin model implemented in the twinlm R function

|  | Variance components | | | Heritability |
| --- | --- | --- | --- | --- |
| Model 1 | $\sigma_{A}^{2}$ = 4.5 | $\sigma_{C}^{2}$ = 8 | $\sigma_{E}^{2}$ = 2.25 | 0.305 |
| Mean | 0.7474 (0.1702) | 1.3267 (0.2058) | 0.3754 (0.0415) | 0.306 (0.0709) |
| Slope | 0.0015 (3e-04) | 0.0027 (4e-04) | 8e-04 (1e-04) | 0.3068 (0.0675) |
|  |  |  |  |  |
| Model 1 | $\sigma_{A}^{2}$ = 10 | $\sigma_{C}^{2}$ = 2.25 | $\sigma_{E}^{2}$ = 2.25 | 0.690 |
| Mean | 1.658 (0.235) | 0.375 (0.228) | 0.3757 (0.0414) | 0.6893 (0.0948) |
| Slope | 0.0033 (4e-04) | 8e-04 (5e-04) | 8e-04 (1e-04) | 0.6911 (0.0918) |
|  |  |  |  |  |
| Model 2c | $\sigma_{A}^{2}$ = 4.3 | $\sigma_{C}^{2}$ = 8 | $\sigma_{E}^{2}$ = 2.25 | 0.296 |
| Mean | 0.7103 (0.1604) | 1.3249 (0.2019) | 0.3758 (0.0416) | 0.2955 (0.068) |
| Slope | 0.0014 (3e-04) | 0.0027 (4e-04) | 8e-04 (1e-04) | 0.2961 (0.0686) |
|  |  |  |  |  |
| Model 2c | $\sigma_{A}^{2}$ = 10 | $\sigma_{C}^{2}$ = 2.25 | $\sigma_{E}^{2}$ = 2.25 | 0.690 |
| Mean | 1.6632 (0.2281) | 0.3664 (0.2297) | 0.3757 (0.0397) | 0.6926 (0.0933) |
| Slope | 0.0033 (5e-04) | 8e-04 (4e-04) | 7e-04 (1e-04) | 0.6915 (0.0926) |

See footnotes for Supplementary Table 2.

**Supplementary Table 4.** Mean (standard deviation) of empirical posterior means for the variance components estimates and heritability estimates from the Bayesian-based approach implemented in WinBUGS

|  | Variance components | | | | | Heritability^*^ |
| --- | --- | --- | --- | --- | --- | --- |
|  | $\sigma_{A}^{2}$ = 4.5 | $\sigma_{C}^{2}$ = 2.25 | $\sigma_{E}^{2}$ = 2.25 | $\tau_{A}^{2}$ = 0 | $\tau_{C}^{2}$ = 0 |  |
| Model 1 | 0.0571  (0.0117) | 0.3417  (0.0911) | 8.2776 (0.2258) | 0 (0) | 0.0003 (0.0001) | 0.0066 (0.0014) |
|  |  |  |  |  |  |  |
|  | $\sigma_{A}^{2}$ = 4.3 | $\sigma_{C}^{2}$ = 2.25 | $\sigma_{E}^{2}$ = 2.25 | $\tau_{A}^{2}$ = 0 | $\tau_{C}^{2}$ = 0 |  |
| Model 2a | 0.0551  (0.0112) | 0.3356  (0.0892) | 8.1003 (0.2156) | 0 (0) | 0.0003 (0.0001) | 0.0065 (0.0013) |
| Model 2b | 0.0552  (0.0113) | 0.3355  (0.0894) | 8.1007 (0.2164) | 0 (0) | 0.0003 (0.0001) | 0.0065 (0.0013) |
| Model 2c | 0.0549  (0.0112) | 0.3369  (0.0899) | 8.1030 (0.2152) | 0 (0) | 0.0003 (0.0001) | 0.0065 (0.0013) |
| Model 2d | 0.0551  (0.0112) | 0.3357  (0.0894) | 8.1004 (0.2158) | 0 (0) | 0.0003 (0.0001) | 0.0065 (0.0013) |
|  |  |  |  |  |  |  |
|  | $\sigma_{A}^{2}$ = 3 | $\sigma_{C}^{2}$ = 1.5 | $\sigma_{E}^{2}$ = 1.5 | $\tau_{A}^{2}$ = 0.001 | $\tau_{C}^{2}$ = 0.001 |  |
| Model 3a | 0.0366  (0.0084) | 0.1208  (0.0547) | 7.4498 (0.2167) | 0 (0) | 0.0008 (0.0001) | 0.0048 (0.0011) |
| Model 3b | 0.0366 (0.0082) | 0.1229  (0.0557) | 7.4429 (0.2135) | 0 (0) | 0.0008 (0.0001) | 0.0048 (0.0011) |
| Model 4a | 0.0246  (0.0056) | 0.0434  (0.0146) | 10.0738 (0.2564) | 0 (0) | 0.0006 (0.0001) | 0.0024  (5e-04) |
| Model 4b | 0.0246  (0.0056) | 0.0434  (0.0147) | 10.0739 (0.2566) | 0 (0) | 0.0006 (0.0001) | 0.0024  (5e-04) |

^*^ Heritability = ${\sigma_{A}^{2}}/{{(\sigma}_{A}^{2}+\sigma_{C}^{2}+\sigma_{E}^{2})}$. “True” heritability resulting from simulation parameters is approximately 0.5 for all models.
